# Models for predicting impact sensitivity of energetic materials based on the trigger linkage hypothesis and Arrhenius kinetics

**DOI:** 10.1007/s00894-019-4269-z

**Published:** 2020-03-04

**Authors:** Tomas L. Jensen, John F. Moxnes, Erik Unneberg, Dennis Christensen

**Affiliations:** Defence Systems Division, Norwegian Defence Research Establishment, P.O. Box 25, N-2027 Kjeller, Norway

**Keywords:** Explosives, Impact sensitivity, Bond dissociation energy, Temperature of detonation, Arrhenius kinetics

## Abstract

In order to predict the impact sensitivity of high explosives, we designed and evaluated several models based on the trigger linkage hypothesis and the Arrhenius equation. To this effect, we calculated the heat of detonation, temperature of detonation, and bond dissociation energy for 70 energetic molecules. The bond dissociation energy divided by the temperature of detonation proved to be a good predictor of the impact sensitivity of nitroaromatics, with a coefficient of determination (*R*^2^) of 0.81. A separate Bayesian analysis gave similar results, taking model complexity into account. For nitramines, there was no relationship between the impact sensitivity and the bond dissociation energy. None of the models studied gave good predictions for the impact sensitivity of liquid nitrate esters. For solid nitrate esters, the bond dissociation energy divided by the temperature of detonation showed promising results (*R*^2^ = 0.85), but since this regression was based on only a few data points, it was discredited when model complexity was accounted for by our Bayesian analysis. Since the temperature of detonation correlated with the impact sensitivity for nitroaromatics, nitramines, and nitrate esters, we consider it to be one of the leading predictive factors of impact sensitivity for energetic materials.

**Electronic supplementary material:**

The online version of this article (10.1007/s00894-019-4269-z) contains supplementary material, which is available to authorized users.

## Introduction

By using quantum chemical and thermodynamic calculations, new energetic molecules can be designed and characterized in terms of their geometry, density, and performance as explosives and propellants [1–5]. It is important to predict the sensitivity of energetic materials, but despite considerable efforts made during the last decades, developing a reliable and general method is still challenging [6–11]. By the sensitivity of an energetic material, we refer to its susceptibility to initiate due to external thermal, mechanical, or electrostatic stimuli. The study of the underlying causes that govern sensitivity is not only important for understanding liquid and solid-state phenomena in general but above all for ensuring safe handling, transport, and storage of energetic materials.

One of the most well-known measures of the sensitivity of an explosive is its impact sensitivity, which is determined by dropping a mass upon the sample, measuring the critical point at which a pre-decided fixed percentage of the drops will lead to an explosion. We refer to this critical point as the *critical impact level* of the material, which may either be given as the critical height (cm) or potential energy (J). Hence, the impact sensitivity and the critical impact level are inversely correlated.

The impact sensitivity is related to macroscopic parameters such as particle size, crystals defects, polymorphism, and crystal orientation. Defects play a particularly prominent role since they form hot-spots under fast compression of the material. The initiation process can be divided into two steps. First, the material is compressed and deformed, leading to heating of the hot-spots. In the second step, the material inside and surrounding the hot-spots self-ignites and propagates into an explosion, provided that the hot-spot temperatures are sufficiently high. The critical temperature at which the explosive self-ignites during impact has been measured to be between 390 and 1060^∘^ C [12, 13].

There are many operational factors which also affect the critical impact level measured, such as the type of fallhammer used, the test procedure itself, and operator-related judgment of explosion/no-explosion. In addition, the impact sensitivity will depend on the thickness and size of the sample [14, 15]. In general, the measured critical impact level depends on a variety of experimental factors in addition to molecular-related properties. Consequently, predicting impact sensitivities with reasonable accuracy appears to be too subtle a problem to be explained by a model based on fallhammer measurements alone.

Additional key factors responsible for the sensitivity of an energetic material include the molecular properties related to the kinetics and the thermodynamics of the decomposition reactions. Numerous studies have been carried out in order to correlate the impact sensitivity with properties like heat of detonation [16–18], detonation velocity [19], bond dissociation energy [22, 23], oxygen balance [24], electrostatic potential of the molecular surface [25–27], band gap [28],^15^N NMR chemical shift [29], “doorway modes” in the region 200–1000 cm^− 1^ [30], and free space in the crystal lattice [31]. More recent studies focusing on physical factors report that there are only weak correlations or trends between the impact sensitivity and heat of detonation, electrostatic potential, and free space in the crystal lattice [7, 8, 17].

Due to the complexity of initiation of the decomposition pointed out by Dlott, care must be taken before drawing mechanistic conclusions based on simple correlation studies [32]. Moreover, if a study is based on too few compounds to make conclusive judgments, we risk asserting accidental correlations [33]. Using a large set of molecules, Keshavarz et al. derived models based on the CHNO ratios and different molecular moieties [34, 35]. Quantitative structure-property relationship (QSPR) models have also been developed for large sets of molecules [36, 37], and seem to be able to predict the impact sensitivity with reasonable accuracy. However, unlike models based on physical factors, these QSPR models do not reveal much information about the intrinsic factors that govern the impact sensitivity. Since they generally contain a surfeit of adjustable parameters, they are also prone to over-fitting.

Models for predicting the impact sensitivity based on the Arrhenius equations and the thermodynamics of the decomposition were introduced in the 1940s and 1950s [12]. In a study of 15 molecules, Wu et al. showed that the ratio between the dissociation energy of the weakest –NO_2_ bond and the heat of decomposition correlated with the impact sensitivity [1, 44]. This approach was refined by Mathieu et al., investigating models based on larger sets of molecules [38–40]. For various families of energetic materials, they found correlations between the impact sensitivity and the bond dissociation energy divided by the decomposition energy. In these models, the enthalpies of formation were either neglected or calculated with a simplified method, and the decomposition energies were computed assuming that the energetic molecule decomposed to H_2_O–CO_2_ arbitrarily [38–40]. Instead of calculating the bond dissociation energies for each molecule separately, the –NO_2_ bond dissociation energy was assigned to a constant value by considering into which family of energetic materials the molecule belonged, along with the functional groups in the neighboring position of the nitro group [38–40].

In this work, we report density functional theory (DFT) calculations of the –NO_2_ bond dissociation energies and thermodynamic calculations of the heat and temperature of detonation for 70 energetic molecules. We then apply our results to investigate how these properties can predict impact sensitivity. All –NO_2_ bond dissociation energies in the molecules are calculated separately. When overlap between the data sets is accounted for, we reach a total of 91 data points on which our regression models are based. Impact sensitivity models based on bond dissociation energies, heats of detonation, detonation temperatures, and total energies are evaluated for these molecules. 1,3,5-Triamino-2,4,6-trinitrobenzene (TATB) is known for its low sensitivity, and has been frequently used in models for predicting impact sensitivities [18, 19, 34, 37, 38]. However, its critical impact level (490 cm;2.5 kg drop weight) is not a measured value but an estimate based on extrapolating the measured critical heights and oxygen balances of only three energetic molecules. The measured critical height of TATB is only reported to be higher than 320 cm [41]. We will therefore use our most promising model to make a more accurate prediction of this value.

## Theory and methods

### Modeling the critical impact level

We model the critical impact level of an energetic mole-cule as a continuous random variable *I* with the property that its (natural) logarithm $\log I$ is governed by a normal distribution with mean *μ* and variance *σ*^2^, so that

1 $$ \log I \sim \mathcal{N}\left( \mu, \sigma^{2}\right). $$

In general, *μ* will depend on the individual choice of molecule, whereas *σ*^2^ is assumed to be constant across families of molecules. The variance will depend on the level of statistical noise in the data set under consideration, which is largely due to experimental inaccuracies. However, when modeling quantum mechanical phenomena, genuine randomness in nature may also have an effect on the measurements. The Bruceton method and UN test procedure are among the most common schemes for measuring critical impact levels. The former gives the impact energy level *I*_50_ (J) or height *H*_50_ (cm) at which 50% of the test samples are expected to explode, whereas the latter gives the impact energy level *I*_1:6_ (J) or height *H*_1:6_ (cm) that results in an explosion for at least one in six test drops [42, 43]. For dimension reasons, we introduce a reference value *I*^0^ of 1 J. We let the critical impact level *I* denote either *I*_50_/*I*^0^ or *I*_1:6_/*I*^0^, depending on whether the data set upon which the regression is based is in accordance with the Bruceton method or the UN test procedure.

We now motivate our choice of models. According to the hot-spot theory, when an energetic material is subjected to a mechanical impact, material deformation will increase the hot-spot temperatures. If this temperature is above a critical level, the molecule will decompose. The trigger linkage hypothesis states that the first step in the initiation of an energetic molecule is a bond cleavage. The decomposition is triggered by the homolytic fission of an A–NO_2_ bond, and so the reaction is given by

2 $$ \text{A--NO}_{2} \rightarrow \left( \text{A--NO}_{2}\right)^{\star} \rightarrow \mathrm{A} \cdot + \cdot \text{NO}_{2}, $$

where the star ⋆ denotes the transition state and A is either C, N, or O.

At high temperatures, similar to what the material is exposed to by impact or shock, the C–NO_2_ bond dissociation is the dominant reaction in the initial decomposition phase for nitroaromatic molecules [33]. However, at lower temperatures, reactions involving the other functional groups on the aromatic ring may occur. Furthermore, auto-catalyzed reactions and self-heating of the material because of exothermal reactions determine the rate of reaction *r* in the next decomposition phase. The kinetic theory attributed to Arrhenius dictates that *r* is inversely exponentially dependent on the activation energy *E*_*a*_ (the energy required to transform the reactant into the transition state), giving us

3 $$ -\frac{\mathrm{d}\left[\text{A--NO}_{2}\right]}{\mathrm{d}t} = r = c\left[\text{A--NO}_{2}\right]^{n}\exp\left( -\frac{E_{a}}{RT}\right), $$

where $\left [\text {A--NO}_{2}\right ]$ is the molar concentration of A–NO_2_, *t* is time, *c* is a constant (the pre-exponential factor), *n* is the reaction order, *R* is the molar gas constant, and *T* is the absolute temperature. Since it has been assumed that the rate of reaction given by the Arrhenius equation correlates negatively with the impact sensitivity of an energetic material [1, 12, 38, 44], we make the assumption that these quantities are inversely proportional. That is, the sum $\mu + \log r$ is constant. Combining this with taking logarithms on both sides of Eq. 3, we get that

4 $$ -\mu = c + n\log\left[\text{A--NO}_{2}\right] - \frac{E_{a}}{RT}. $$

When $\left [\text {A--NO}_{2}\right ]$ and *n* are assumed to be constant, Eq. 4 takes the form

5 $$ \mu = c_{1} + \frac{E_{a}}{RT}, $$

where *c*_1_ is a constant.

Equation 5 forms the basis for our models. In order to calculate *μ*, we first need to calculate *E*_*a*_ and *T*. Unfortunately, these parameters are difficult to determine. Even though *E*_*a*_ can be calculated by quantum mechanical methods, several transition states and different decomposition routes need to be considered, making the calculations very time-consuming. For this reason, we have evaluated various approximation schemes for *E*_*a*_ and *T*. In our first three models, we assume that the activation energy in Eq. 5 is constant.

The Arrhenius law requires a particular temperature. During the complex sequence of events leading to an explosion, the temperature in the surroundings of a decomposed molecule will deviate from the ambient temperature. A small number of neighboring molecules are envisioned to decompose and release energy, increasing the local temperature. If the decomposition reaction produces more heat than is lost in conjunction with the heating of the nearby species, heat convection, and conduction to the surroundings, the temperature will rise rapidly. If this occurs, the reaction may propagate into an explosion. Therefore, *T* is a local temperature, varying in space and time during these events. In this picture, the more energy released during decomposition, the higher the local temperature. Consequently, the heat of detonation *Q* (kJ dm^− 3^) is assumed to be proportional to *T*. This gives our first model, which was studied in References [1, 7, 8, 16–18, 44], namely

6 $$ \mu = c_{1} + \frac{c_{2}}Q. $$

Here, the constants *c*_1_, *c*_2_ are fitted to critical impact level data by linear regression and *Q* is calculated in EXPLO5 [45], which uses the chemical formula, the enthalpy of formation, and density to calculate the detonation properties. The enthalpies of formation and densities are taken from References [46–49]. These references do not contain enthalpy of formation or density for all the energetic materials we shall consider, so for the remaining molecules, these parameters are estimated using the method described in References [50–54].

Song et al. assumed that the total energy *E*_total_ (Hartree) of an energetic molecule correlates with the energy release of the decomposition reaction [20, 21]. This motivates the assumption that *E*_total_ and *T* are proportional, giving us the model

7 $$ \mu = c_{1} + \frac{c_{2}}{E_{\text{total}}}, $$

where *E*_total_ is calculated by Gaussian09 [55]. The zero-point energy is not included in this calculation.

A new model, to our knowledge not discussed in the literature, results from assuming that *T* in Eq. 5 is proportional to the detonation temperature *T*_ex_ (K) of the material, leading us to

8 $$ \mu = c_{1} + \frac{c_{2}}{T_{\text{\scriptsize ex}}}, $$

where *T*_ex_ is calculated in EXPLO5.

The models above require a constant *E*_*a*_ for the homolytic cleavage of the A–NO_2_ bond for each class of energetic material. This is clearly a rough approximation. It has been proposed that *E*_*a*_ is proportional to the bond dissociation energy BDE (kJ mol^− 1^), that is, the energy required to break the trigger-linkage [1, 38–40, 44]. However, a proportional relationship between BDE and *E*_*a*_ is likely to hold only for compounds where the resonance stabilization and the structure of the transition states are relatively similar. Khrapkovskii et al. reported a significant correlation between the measured value of *E*_*a*_ for the C–NO_2_ homolysis in nitroaromatic substances with different substituents and the values of BDE calculated by the hybrid DFT functional B3LYP and a small basis set 6-31G(d,p), with a coefficient of determination *R*^2^ of 0.72 [56]. This motivates the assumption that *E*_*a*_ and BDE are proportional.

Our fourth model in question was studied in References [22, 23]. It is based on BDE alone, and reads

9 $$ \mu = c_{1} + c_{2}\left( \text{BDE}\right), $$

where we have assumed that *T* in Eq. 5 is constant. By including BDE and the approximations used in Eqs. 6, 7, and 8 for the local temperature into Eq. 5, we arrive at our final three models of consideration, which take the form

10 $$ \begin{array}{@{}rcl@{}} \mu & =& c_{1} + c_{2}\left( \frac{\text{BDE}}Q\right), \end{array} $$

11 $$ \begin{array}{@{}rcl@{}} \mu & = &c_{1} + c_{2}\left( \frac{\text{BDE}}{E_{\text{total}}}\right), \end{array} $$

12 $$ \begin{array}{@{}rcl@{}} \mu & = &c_{1} + c_{2}\left( \frac{\text{BDE}}{T_{\text{\scriptsize ex}}}\right). \end{array} $$

Equations 10 and 11 were studied in References [1, 19, 38–40, 44] and [20, 21], respectively.

We study three families of energetic molecules: nitroaromatics, nitramines, and nitrate esters. For each family, we make a choice of which bond rupture we believe to be the key step in the initiation process. We choose C–NO_2_, N–NO_2_, and O–NO_2_ for nitroaromatics, nitramines, and nitrate esters, respectively.

### Density functional theory calculations

Our original intention was to optimize the geometry of molecules and radicals with the M06 functional and the 6-311+G(2d,p) basis set, since M06 is reported to calculate homolytic dissociation of C–NO_2_ bonds accurately [57]. However, we were not able to calculate several hundred values of BDE with this choice of functional and basis set due to limited computer power. In order to avoid this difficulty, we instead chose the B3LYP functional, which is widely used in optimizing the geometry of energetic materials. This functional is known to systematically undershoot the value of BDE for C–NO_2_ [57], but Khrapkovskii et al. have shown that with a small basis set (6-31G(d,p)), it calculates BDE for substituted nitroaromatics with similar accuracy as wB97xd/6-31+G(2df,p), G2, G3, G3B3 and CBS-QB3 [56]. In Table 1, we show how the calculated value of the C–NO_2_ BDE for nitrobenzene converges by increasing the size of the basis set. This value has been measured to be 298.7 kJ mol^− 1^ [58] and 314.5 kJ mol^− 1^ [59].

**Table 1:** The C–NO_2_ bond dissociation energy BDE (kJ mol^− 1^) for nitrobenzene calculated with the B3LYP and M06 functional at 298 K by using different basis sets

Basis set	B3LYP	M06
6-31G	301.3	329.9
6-31G(d)	290.1	312.6
6-31G(d,p)	290.2	312.6
6-311G(d,p)	278.7	299.7
6-31+G(d,p)	282.6	305.4
6-311+G(d,p)	276.3	297.5
6-311+G(2df,2p)	277.3	299.6

The BDE values in Table 1 are calculated according to the method in Reference [60]. Table 1 also illustrates how the B3LYP functional undershoots the BDE values, but it should be borne in mind that in our models, differences in BDE are more important than the particular values they take. The calculation is defined by

13 $$ \text{BDE} = E\left( \mathrm{A}\cdot\right) + E\left( \text{NO}_{2}\cdot\right) - E\left( \text{A--NO}_{2}\right), $$

where $E\left (\mathrm {A}\cdot \right ), E\left (\text {NO}_{2}\cdot \right )$, and $E\left (\text {A--NO}_{2}\right )$ denote the ground state electronic energies (open shell model) of the species A⋅,NO_2_ ⋅ and A–NO_2_, respectively. In Eq. 13, the zero-point energy is neglected since Song et al. have shown that it bears no important role for the correlation between *I*_50_ and BDE/*E*_total_ [20].

### Experimental measurements

Since variation in the measurements of critical impact level causes difficulties in parametrizing and validating models, it is important to keep observational uncertainties to a minimum when conducting experiments. The critical impact level data for the nitroaromatic materials is obtained from the Wilson et al. data set, where the tests were performed in the same laboratory with the same equipment and according to the same test procedure [48]. Wilson et al. also ensured that the molecules considered had a similar particle distribution, and so we find this data set to be the most useful one for our purposes. It should be noted that the critical height of 1,3-diamino-2,4,6-trinitrobenzene (DATB) and TATB are only given as a lower limit below which they did not explode, namely *H*_50_ > 200 cm for both molecules. Therefore, these values are not included in the training set from Wilson et al. In our analysis, we also consider two other data sets for nitroaromatics in order to reduce the risk of asserting any accidental correlations. These are taken from Storm et al. [41] and Meyer et al. [47]. The critical impact levels for the nitramines and nitrate esters are obtained from Storm et al. and Meyer et al., respectively. For the Wilson et al. and Storm et al. data sets, the Bruceton procedure was used, while the Meyer et al. data set is based on the UN test procedure. Hence, there are systematic differences between the measured critical impact levels in these data sets, which emphasizes that we cannot easily combine them in order to parametrize and validate the models collectively [8].

### Statistical analysis

In order to evaluate the predictive ability of our models, we calculate the coefficient of determination (*R*^2^), root-mean-square error (RMSE), absolute mean, and maximum deviation between our predictions and the measurements from our data sets. The most promising model is also developed in parallel via Bayesian regression, taking model complexity into account. We evaluate the predictive power of this Bayesian model via the model evidence function. In addition, we also perform a simple sensitivity analysis in order to evaluate the consequences of inaccurate calculations or measurements.

In the frequentist framework, the mean *μ* and variance *σ*^2^ in the distribution (1) are estimated using the sample mean and correctly scaled sample variance, respectively, which are unbiased. We also note that our assumption (1) may be rephrased as

14 $$ I \sim \text{Lognormal}\left( \mu, \sigma^{2}\right), $$

and so in the frequentist framework, we may predict the mean and variance of a new critical impact level *I*_new_ as

15 $$ \begin{array}{@{}rcl@{}} \mathbb{E}\left[I_{\text{\scriptsize new}}\right] & =& \exp\left( \mu + \frac{\sigma^{2}}2\right), \end{array} $$

16 $$ \begin{array}{@{}rcl@{}} \text{var}(I_{\text{\scriptsize new}}) & =& \left[\exp\left( \sigma^{2}\right)-1\right]\exp\left( 2\mu + \sigma^{2}\right). \end{array} $$

In the Bayesian framework, we introduce a conjugate prior distribution for the model coefficients and estimate the variance by maximizing the evidence function. Then any new critical impact level $I_{\text {\scriptsize new}}|\mathcal {D}$ given observed data $\mathcal {D}$ will be governed by the predictive distribution, which will also be lognormal due to the functional form of the prior distribution. That is,

17 $$ I_{\text{\scriptsize new}}|\mathcal{D} \sim \text{Lognormal}\left( \mu_{\mathcal{D}}, \sigma^{2}_{\mathcal{D}}\right), $$

where $\mu _{\mathcal {D}}$ and $\sigma ^{2}_{\mathcal {D}}$ are the mean and variance of the predictive distribution, respectively. Recall that unlike in the frequentist framework, $\sigma ^{2}_{\mathcal {D}}$ will depend on the individual choice of molecule considered. The expectation and variance of the new critical impact level $I_{\text {\scriptsize new}}|\mathcal {D}$ may then be calculated as

18 $$ \begin{array}{@{}rcl@{}} \mathbb{E}\left[I_{\text{\scriptsize new}}|\mathcal{D}\right] & =& \exp\left( \mu_{\mathcal{D}} + \frac{\sigma^{2}_{\mathcal{D}}}2\right), \end{array} $$

19 $$ \begin{array}{@{}rcl@{}} \text{var}\left( I_{\text{\scriptsize new}}|\mathcal{D}\right) & =& \left[\exp\left( \sigma^{2}_{\mathcal{D}}\right) - 1\right]\exp\left( 2\mu_{\mathcal{D}} + \sigma^{2}_{\mathcal{D}}\right). \end{array} $$

Later, we predict the critical impact level for DATB and TATB, both in the frequentist and Bayesian framework.

## Results and discussion

The molecular structure of these energetic materials together with the computed data are available in the Electronic Supplementary Material.

### Nitroaromatics

We perform the linear regression for nitroaromatic materials in two separate ways, both in a frequentist framework and via Bayesian regression. In the former framework, the model coefficients are determined by minimizing a non-regularized sum-of-squares error function, and the predictive ability of the model is evaluated via cross-validation. Note that with this approach, the issue of model complexity is not addressed. However, when performing regression with relatively few data points, the issue of model complexity becomes a key point for avoiding over-fitting. In the Bayesian framework, analysis of model complexity is built in by design, which leads us to somewhat different conclusions than those relying on the frequentist approach, in particular for the Storm et al. data set. In the Bayesian framework, the predictive quality of the model is addressed by evaluating the model evidence rather than cross-validation.

#### Frequentist framework

The results for the nitroaromatic materials based on the Wilson et al., Storm et al., and Meyer et al. data sets are shown in Table 2.

**Table 2:** The coefficient of determination between the log critical impact level and the reciprocal of the heat of detonation, the reciprocal of the total energy, the reciprocal of the temperature of detonation, the bond dissociation energy, the bond dissociation energy divided by the total energy, the bond dissociation energy divided by the heat of detonation, and the bond dissociation divided by the temperature of detonation. The regression is based on the Wilson et al., Storm et al., and Meyer et al. data sets, respectively

	1/*Q*	1/*E*_total_	1/*T*_ex_	BDE	BDE/*E*_total_	BDE/*Q*	BDE/*T*_ex_
Data set	(dm^3^ kJ^− 1^)	($E_{\text {h}}^{-1}$)	(K^− 1^)	(kJ mol^− 1^)	(kJ mol$^{-1} E_{\text {h}}^{-1}$)	(dm^3^ mol^− 1^)	(kJ mol^− 1^ K^− 1^)
Wilson et al.	0.20	0.24	0.41	0.56	0.48	0.76	0.81
Storm et al.	0.41	0.26	0.54	0.56	0.42	0.64	0.67
Meyer et al.	0.64	0.21	0.75	0.41	0.40	0.70	0.69

**Fig. 1:**
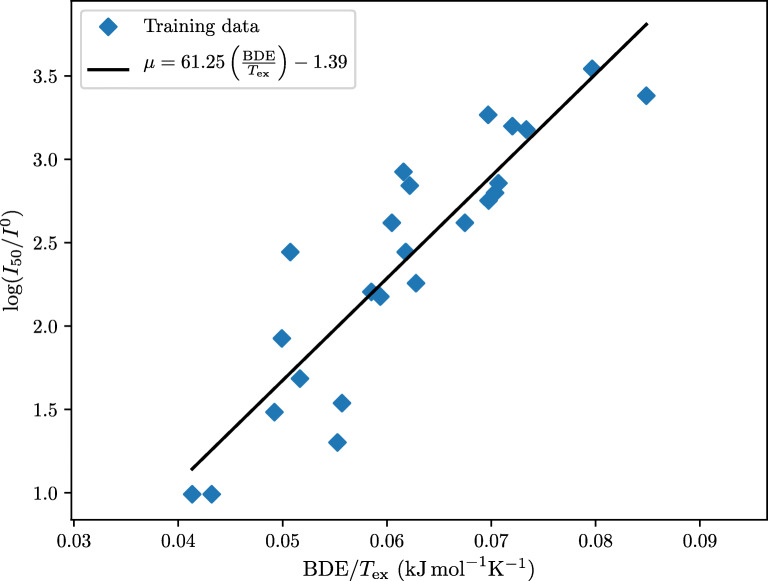
The log critical impact level of the nitroaromatics in the Wilson et al. data set plotted against the bond dissociation energy divided by the detonation temperature, along with the best-fitting regression line (*R*^2^ = 0.81)

We note that 1/*T*_ex_ correlates better with $\log I$ than 1/*Q* for all three data sets. The most promising predictor of critical impact levels overall is BDE/*T*_ex_, with *R*^2^ = 0.81,0.67, and 0.69. Figure 1 illustrates the merit of this model for the Wilson et al. data set.

Table 2 shows only a weak correlation between BDE/*E*_total_ and $\log I$. These results are not in line with those of Song et al., which indicate merit for this model [20, 21]. However, they derived this correlation by using a small data set. For molecules of similar structure, *E*_total_ is likely to correlate with *Q*, but this is unlikely to hold in general; we get virtually no correlation (*R*^2^ = 0.03) when plotting these parameters against each other.

As there are only 24, 17, and 16 molecules in the Wilson et al., Storm et al., and Meyer et al. data sets, respectively, our regression is a priori prone to over-fitting. In order to evaluate its predictive ability, we use leave-one-out cross-validation; the results of which are summarized in Table 3.

**Table 3:** Leave-one-out cross-validation of the model based on the bond dissociation energy divided by the temperature of detonation. The RMSE, absolute average deviation, maximum deviation, and the compounds with largest deviation are given. See the “Statistical analysis” section for how the predicted critical impact level is calculated from the model

		Average abs.	Maximum	Maximum
Data set	RMSE (J)	deviation (J)	deviation (J)	deviation compound
Wilson et al.	6.1	3.7	25	CL-14
Storm et al.	15	10	40	Styphnic acid
Meyer et al.	9.1	6.6	21	Picramic acid

Our cross-validation does not show any particularly convincing results for either data set, revealing that more parameters than just BDE/*T*_ex_ are needed to predict the critical impact level of nitroaromatic compounds. The most promising numbers are for the Wilson et al. data set, with an RMSE of 6.1 J and an average absolute deviation of 3.7 J between the predicted and measured *I*_50_. 5,7-Diamino-4,6-dinitrobenzofuroxan (CL-14) has the largest deviation (25 J). The (Wilson et al.) model predicts the critical impact level *I*_50_ of CL-14 to be 48 J, while the measured *I*_50_ is 29 J. We note that the calculated C–NO_2_ BDE in CL-14 is quite large, at 318 kJ mol^− 1^. However, this NO_2_ group is surrounded by an amino group on each of the neighboring carbons, as is also the case for TATB. Hence, this value for CL-14 is not particularly surprising, as the BDE of TATB is calculated to be 310 kJ mol^− 1^. The deviation between the measured and predicted critical impact level of CL-14 may indicate that this molecule follows another decomposition route.

CL-14 contains a furoxan ring, as is also the case for three other molecules in the Wilson et al. data set, namely 7-amino-4,6-dinitrobenzofuroxan (ADNBF), 4,6-dinitrobenzofuroxan (DNBF), and 8-amino-7-nitrobenzobisfuroxan (CL-18). These have deviations of -3 J, -8 J, and 2 J (respectively) between the predicted and measured *I*_50_. If the decomposition is initiated in the furoxan ring, a larger deviation may be expected.

From Table 3, we see that in the Storm et al. data set, 1,3-dihydroxy-2,4,6-trinitrobenzene (styphnic acid) has the largest deviation between the predicted and measured *I*_50_. Our model predicts this value to be 45 J, while it is measured to be 11 J. The BDE is calculated to be 287 kJ mol^− 1^ by using the M06 functional and the 6-311G+(2d,p) basis set. This is similar to the value calculated when using B3LYP/6-31G(d), which is 274 kJ mol^− 1^.

#### Bayesian regression

We now perform Bayesian linear regression separately on the three data sets for the most promising model, namely (12). In order to adapt the Bayesian framework, we introduce a zero-mean, isotropic bivariate normal prior distribution over the model coefficients **c** = (*c*_1_, *c*_2_)^*T*^ with covariance matrix *τ*^2^*I*, so that

20 $$ \mathbf{c} \sim \mathcal{N}\left( \mathbf{0}, \tau^{2}I\right). $$

We follow the process outlined in Reference [61], in which the first step is to maximize the evidence function in order to obtain estimates for the parameters *σ*^2^ and *τ*^2^, a technique also known as emperical Bayes. We then compute the posterior distributions for the model coefficients by updating our prior distributions separately over the the data sets. Using the mean of this posterior distribution as our estimate for the model coefficients **c**, we obtain our linear models. Note that this process is equivalent to minimizing a regularized sum-of-squares error function with regularization term *λ* = *σ*^2^/*τ*^2^, and so model complexity is intrinsically accounted for. A summary of the model coefficients computed, along with the relevant parameters, is provided in Table 4.

**Table 4:** Bayesian regression for the three nitroaromatic data sets, based on Eq. 12, with a prior distribution given by Eq. 20. The variances *σ*^2^ and *τ*^2^, the regularization coefficient *λ* = *σ*^2^/*τ*^2^ and the effective number of parameters *γ* = **c**^*T*^**c**/*τ*^2^ are also included

Data set	*c*_1_	*c*_2_	*σ*^2^	*τ*^2^	*λ* = *σ*^2^/*τ*^2^	*γ* = **c**^*T*^**c**/*τ*^2^
Wilson et al.	− 1.31	59.92	0.11	1.7 × 10^3^	6.2 × 10^− 5^	1.98
Storm et al.	2.80	2.18	0.82	12	6.7 × 10^− 3^	1.03
Meyer et al.	− 0.81	44.40	0.23	1.0 × 10^3^	2.2 × 10^− 4^	1.93

We see that the penalizing regularization term plays a substantial role for the regression on the Storm et al. data set. Although values of *R*^2^ in the frequentist analysis for Storm et al. and Meyer et al. were relatively close in value (0.67 and 0.69, respectively), the Bayesian analysis suggests that there is little evidence supporting a linear term in the model for the Storm et al. data set. Considering the interpretation of the parameter *γ* = **c**^*T*^**c**/*τ*^2^ provided in Reference [62], namely as the *effective number of parameters* for the model, we see that for the Storm et al. data set, there is effectively only a single parameter (the constant term) governing the distribution of data points. This is in contrast to the corresponding results for the Wilson et al. and Meyer et al. data sets, where the effective number of parameters is calculated to be approximately equal to 2, supporting the claim that $\log I$ indeed depends linearly on BDE/*T*_ex_.

We evaluate the predictive ability of our models in the Bayesian framework by calculating the ($\log $) model evidence function. Since the Bayesian regression penalizes the model complexity for the Storm et al. data set, we also compare the evidence for our proposed model to that of a separate constant model which asserts no correlation between $\log I$ and BDE/*T*_ex_. That is, our other model claims that *I* is governed by a lognormal distribution of the form

21 $$ I \sim \text{Lognormal}\left( \mu_{0}, {\sigma_{0}^{2}}\right) $$

with constant mean *μ*_0_ and constant variance ${\sigma _{0}^{2}}$. We perform the corresponding Bayesian regression for this alternative model and compare the results to our original model (12) by calculating the Bayes factor. The results of this model comparison are presented in Table 5. A Bayesian factor larger than unity indicates preference towards the linear model, while a factor smaller than unity indicates preference towards the constant model.

**Table 5:** Evaluation of the (log) model evidence for the proposed linear model (12), along with the (log) model evidence for an alternative constant model (21). The Bayes factor and its preference are also listed for the three data sets

	Linear hypothesis	Constant hypothesis	Bayes factor	Preferred
Data set	model evidence (log)	model evidence (log)	(linear against constant)	model
Wilson et al.	− 14.46	− 26.13	1.2 × 10^5^	Linear
Storm et al.	− 23.40	− 21.12	0.10	Constant
Meyer et al.	− 17.78	− 18.96	3.25	Linear

From Table 5, we see that Bayesian regression on the Wilson et al. and Meyer et al. data sets prefers the linear model (12), whereas it prefers the constant model (21) for the Storm et al. data set. Hence, our prediction of the critical impact level of TATB will be based on the Wilson et al. data set rather than that of Storm et al. Note that the model comparison for the Wilson et al. data set is several orders of magnitude more decisive than for that of Meyer et al.

We make two predictions of the critical impact level *I*_50_ of DATB and TATB, one based on the frequentist framework and another from the Bayesian predictive distribution. The predictions and their uncertainties are calculated using Eqs. 15 and 16. The results are given in Table 6.

**Table 6:** Frequentist and Bayesian predictions of the critical impact level *I*_50_ (J) of DATB and TATB based on Eq. 12. The standard deviation of the respective predictions are included in parentheses

					Frequentist	Bayesian
	*μ*	$\mu _{\mathcal {D}}$	*σ*^2^	$\sigma ^{2}_{\mathcal {D}}$	predicted *I*_50_ (J)	predicted *I*_50_ (J)
DATB	4.06	4.03	0.11	0.15	61 (21)	60 (24)
TATB	4.52	4.48	0.11	0.16	97 (34)	95 (40)

**Fig. 2:**
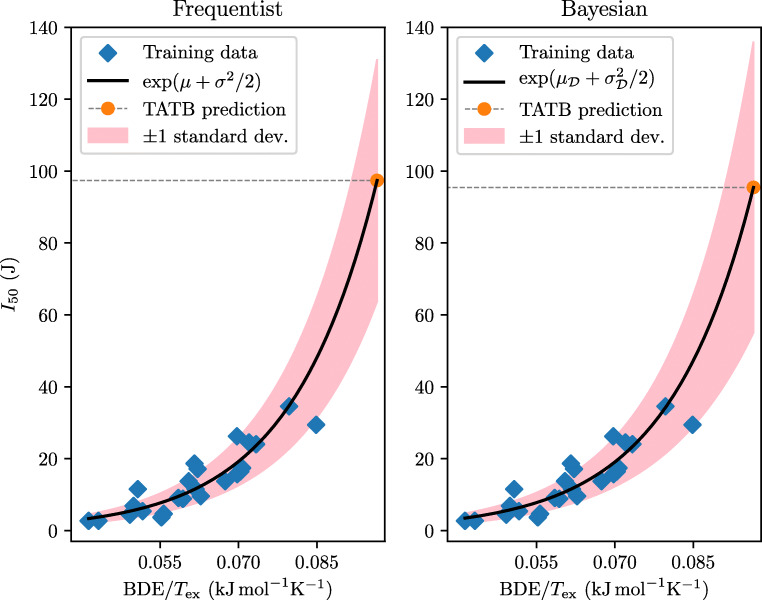
Frequentist and Bayesian predictions of the critical impact level of TATB, based on the lognormal model fitted to the Wilson et al. data set. The red-shaded region comprises one standard deviation on either side of the exponential curve, as calculated by Eqs. 16 and 19 for the frequentist and Bayesian prediction, respectively

Storm et al. measured the critical impact level (*H*_50_, 2.5 kg drop weight) of TATB to be larger than 320 cm (*I*_50_ > 78 J). Additionally, they predicted *H*_50_ to be 490 cm (*I*_50_ = 120 J) based on the measured impact sensitivity and the oxygen balance for trinitrobenzene, 2,4,6-trinitroaniline and DATB [41]. Using the Wilson et al. data set, our frequentist and Bayesian prediction of*I*_50_ for TATB are 97 J and 95 J, respectively. We note that the Bayesian prediction is less certain since the variance of the predictive distribution accounts for uncertainty related to the model coefficients, as well as noise in the data. This difference in certainty for the two models is illustrated in Fig. 2.

The frequentist and Bayesian predictions of *I*_50_ for DATB are 61 J and 60 J, respectively. In the Storm et al. data set, its measured value is 78 J [41], which is consistent with the difference between the Wilson et al. data set and the Storm et al. data set. Indeed, the former data set is more sensitive than the latter for materials with a critical impact level larger than 15 J. For more sensitive materials (*I*_50_ < 15 J), the differences in critical impact level are smaller [8]. In the Storm et al. data set, a sample mass of 40 mg was used, while in the Wilson et al. data set, it was 35 mg. It has been reported that nitroaromatics such as tetryl, 2,4,6-trinitrotoluene (TNT) and o-trinitrophenol become more sensitive when the thickness or the amount of sample is reduced [14, 15]. The smaller the sample, the more energy is released per unit volume of explosive. The Wilson et al. data set is based on a smaller sample mass than that used in the Storm et al. data set, which may be one of the main reasons for the difference in measured critical impact levels.

#### Model sensitivity analysis

We now perform a simple sensitivity analysis of our frequentist and Bayesian models, investigating how sensitive they are to slight changes in the input variables upon which the regression is based. From the “Bayesian regression” section, we expect the Bayesian model to be more sensitive to such alterations than the frequentist model, since the variance of the Bayesian predictive distribution also accounts for uncertainty in the model coefficients. This can be seen explicitly by observing how the predicted value of *I*_50_ for DATB changes if we over- and underestimate the values of BDE and *T*_ex_ in turn in the Wilson et al. data set. When multiplying BDE and *T*_ex_ by (1 + *ε*), where *ε* = -0.05,-$0.04, \dots , 0.04, 0.05$, the frequentist prediction of *I*_50_ for DATB changes by no more than 10^− 13^% of its original value. On the other hand, the corresponding difference in the Bayesian prediction ranges from 10^− 8^% to 10^− 6^%.

Inaccuracies in the measured critical impact level may also affect how the model makes new predictions. We measure the sensitivity of our frequentist and Bayesian models by multiplying the values of *I* in the data set by (1 + *ε*), where *ε* is drawn (separately for each molecule) from a uniform probability distribution of range [-0.05,0.05]. We then calculate the absolute difference in the predicted *I*_50_ of DATB before and after perturbing the input, given as a percentage of the original predicted value of *I*_50_. Averaging over 500 such random simulations, we find the predicted *I*_50_ of DATB to deviate from the original prediction by a factor of 1.3% for both the frequentist and the Bayesian model. This result indicates that inexact experimental measurements may sully the accuracy of the model.

### Nitramines

The results for the nitramines based on the Storm et al. data set are shown in Table 7. We see that neither 1/*Q* nor 1/*T*_ex_ is strongly correlated with $\log I$ (*R*^2^ = 0.34 and 0.44, respectively), and for the N–NO_2_ BDE, we get virtually no correlation with $\log I$ (*R*^2^ = 0.04).

**Table 7:** The coefficient of determination (*R*^2^) between the log critical impact level of nitramine compounds and the reciprocal of the heat of detonation, the reciprocal of the total energy, the reciprocal of the temperature of detonation, the bond dissociation energy, the bond dissociation energy divided by the total energy, the bond dissociation energy divided by the heat of detonation, and the bond dissociation divided by the temperature of detonation. The calculations are first based on using the weakest (N-N) bond, and then the weakest C–NO_2_ or N–NO_2_ bond. The regression is based on the Storm et al. data set

	1/*Q*	1/*E*_total_	1/*T*_ex_	BDE	BDE/*E*_total_	BDE/*Q*	BDE/*T*_ex_
Data set	(dm^3^ kJ^− 1^)	($E_{\text {h}}^{-1}$)	(K^− 1^)	(kJ mol^− 1^)	(kJ mol$^{-1} E_{\text {h}}^{-1}$)	(dm^3^ mol^− 1^)	(kJ mol^− 1^ K^− 1^)
N–NO_2_	0.34	0.12	0.44	0.04	0.10	0.41	0.34
C–NO_2_ or N–NO_2_	0.34	0.12	0.44	0.11	0.12	0.49	0.41

**Fig. 3:**
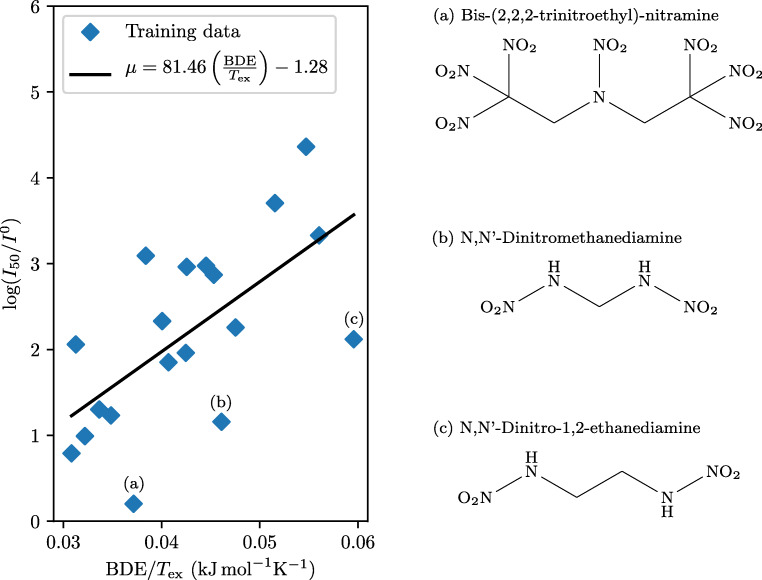
The log critical impact level of the nitramines in the Storm et al. data set plotted against the bond dissociation energy divided by the detonation temperature, along with the best-fitting linear regression line (*R*^2^ = 0.41). The weakest N–NO_2_ or C–NO_2_ bond is used as the weakest bond for the model

In addition to N–NO_2_ bond, some of the nitramines contain one or more C–NO_2_ bonds. For such molecules, the C–NO_2_ bond may be weaker than the N–NO_2_ bond when three NO_2_ groups are attached to the same carbon atom. This is due to repulsive forces between the NO_2_ groups and also their attraction to electrons in the C–NO_2_ bond. If the weakest bond in the model is taken to be either N–NO_2_ or C–NO_2_, there is still low correlation between $\log I$ and BDE, indicating that molecular rearrangements and auto-catalyzed reactions play a key role in the initial nitramine decomposition. Contrary to the promising result for nitroaromatics, we only see a weak correlation between $\log I$ and BDE/*T*_ex_ (*R*^2^ = 0.41). This observation is illustrated in Fig. 3, where $\log I$ is plotted against BDE/*T*_ex_.

We now comment on the outliers marked in Fig. 3. First, bis-(2,2,2-trinitroethyl)-nitramine has a positive oxygen balance which results in a relatively low value of *T*_ex_ since the molecule does not contain enough carbon or hydrogen to utilize all the oxygen. This leads to a particularly high value of BDE/*T*_ex_, which illustrates how our model predicts compounds with a positive oxygen balance to be less sensitive to impact than what they actually are. Next, N,N’-dinitromethanediamine and N,N’-dinitro-1,2-ethanediamine contain the –NH–NO_2_ functional group. The values of the N–NO_2_ BDE for these molecules (220 kJ mol^− 1^ and 214 kJ mol^− 1^, respectively) are relatively high compared with those of nitramines, which usually range from 150 to 170 kJ mol^− 1^. We calculated BDE of these compounds with the M06 functional and the 6-311+G(2d,p) basis set in order to exclude the possibility for any erroneous geometry optimization caused by the B3LYP functional. The M06 functional with the 6-311+G(2d,p) basis set predicts BDE for N,N’-dinitromethanediamine and N,N’-dinitro-1,2-ethanediamine to be 244 kJ mol^− 1^ and 241 kJ mol^− 1^, respectively. Thus, the divergence of these two molecules from the regression line cannot be explained by the high BDE calculated by the B3LYP functional alone.

The initial decomposition of nitramines can take place through several mechanistic routes. For example, at least four initial mechanisms for the decomposition of 1,3,5-trinitroperhydro-1,3,5-triazine (RDX) have been suggested: N–NO_2_ homolysis, HONO elimination, the “tripple whammy” mechanism, and NONO isomerization. Using the couple cluster theory, the *E*_*a*_ value of the HONO elimination has been calculated to be lower than that of the N–NO_2_ homolytic reaction for RDX [63]. A recent study of the initial decomposition process of liquid RDX has revealed that HONO elimination is likely to be the major decomposition pathway [64].

In order to investigate whether HONO elimination is an alternative decomposition route for N,N’-dinitromethanediamine, we calculate the energy required to break the N–NOOH bond. The HONO BDE is calculated to be 420 kJ mol^− 1^. When the hydrogen atom is moved from the nitrogen atom to the oxygen atom, the length of the N–N bond decreased from 1.379 to 1.257 Å (M06/6-311+G(2d,p)). The bond becomes more like a double bond, making it unlikely to break without more molecular rearrangements.

### Nitrate esters

The nitrate ester data set (from Meyer et al.) consists of both liquids and solids. Table 8 shows that when these are considered simultaneously, none of our models seems to give any satisfactory predictions. When solids and liquids are treated separately, no model shows any notable improvement for the liquids. Denisaev et al. have found a correlation between $\sqrt {H_{50}}$ and *ρQ*, where *ρ* is the density, for liquid nitrate esters [16]. The critical impact levels of liquid nitrate esters are sensitive to experimental factors such as the presence of bubbles in the liquid which can significantly alter the impact sensitivity of a liquid explosive [12].

**Table 8:** The coefficient of determination (*R*^2^) between the log critical impact level of nitrate ester compounds and heat of detonation, total energy, detonation temperature, and bond dissociation energy and ratios between the bond dissociation energy and heat of detonation, total energy, and detonation temperature. The regression is based on the Meyer et al. data set

	1/*Q*	1/*E*_total_	1/*T*_ex_	BDE	BDE/*E*_total_	BDE/*Q*	BDE/*T*_ex_
Data set	(dm^3^ kJ^− 1^)	($E_{\text {h}}^{-1}$)	(K^− 1^)	(kJ mol^− 1^)	(kJ mol$^{-1} E_{\text {h}}^{-1}$)	(dm^3^ mol^− 1^)	(kJ mol^− 1^ K^− 1^)
Solids + liquids	0.00	0.19	0.17	0.00	0.19	0.00	0.11
Solids	0.29	0.83	0.83	0.68	0.49	0.49	0.85
Liquids	0.05	0.16	0.16	-0.02	0.11	0.03	0.10

**Fig. 4:**
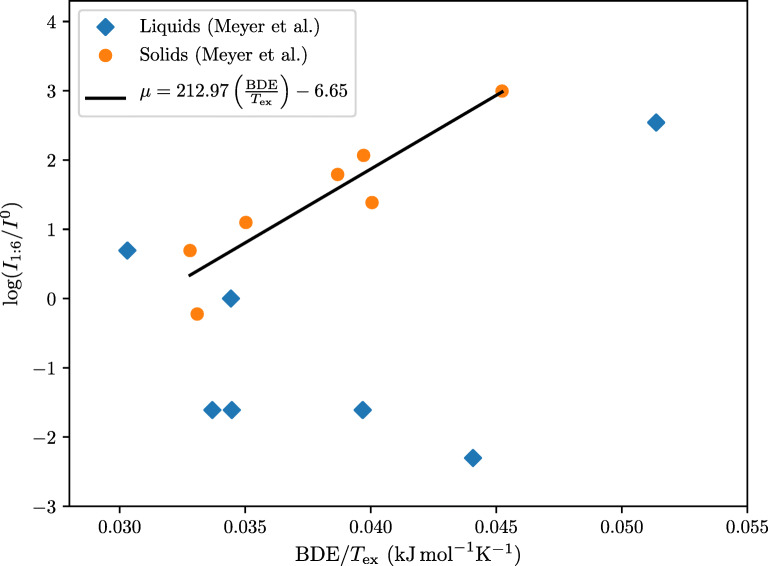
The log critical impact level of the liquid and solid nitrate esters in the Meyer et al. data set plotted against the bond dissociation energy divided by the detonation temperature, along with the best-fitting linear regression line for the solids (*R*^2^ = 0.85)

There seems to be a strong correlation between $\log I$ and BDE/*T*_ex_ for solid nitrate esters, but as can be seen in Fig. 4, this assertion is based on very few data points. In order to investigate whether this correlation is genuine or just a result of over-fitting, we perform a Bayesian model comparison with a constant model (which asserts no correlation between BDE/*T*_ex_ and $\log I$) as in the “Bayesian regression” section. When model complexity is accounted for, the Bayes factor comes out to be 0.554, in favor of the constant model. Hence, more data points are needed for further investigation of the the merit of the linear model. Similar results hold for the other seemingly promising model, namely (8), with a Bayes factor of 0.552, again favoring the constant model.

## Conclusion

By investigating 70 energetic nitroaromatics, nitramines, and nitrate esters, we have evaluated seven models for predicting critical impact level, the quantity from which impact sensitivity is determined. Input parameters were the molecules’ temperature of detonation, heat of detonation, and bond dissociation energy. Our regression was based on three separate data sets comprising 91 data points in total.

For the largest nitroaromatics data set, the bond dissociation energy divided by the temperature of detonation was the best predictor of critical impact level, with a coefficient of determination (*R*^2^) of 0.81. Leave-one-out cross-validation gave a root-mean-square error (RMSE) of 6.1 J and the absolute average deviation was 3.7 J between the predicted and the measured values. A separate Bayesian regression also assigned similar merit to the predictive power of this model, also when accounting for model complexity. The frequentist and Bayesian models predicted the critical impact level of TATB to be 97 J and 95 J, respectively. Our sensitivity analysis showed this prediction to be more robust under changes in calculations of molecular parameters than measurements of critical impact levels.

For nitramines, our analysis showed that the temperature of detonation is a moderately useful predictor of critical impact level, unlike the N–NO_2_ bond dissocation energy, which we found to have virtually no such predictive ability. Hence, N–NO_2_ homolysis is unlikely to be the only reaction taking place in the initial phase of the decomposition.

None of the models was able to predict the critical impact level of liquid nitrate esters, but for a small data set of solid nitrate esters, there seemed to be promising results for bond dissociation energy divided by the temperature of detonation. However, our Bayesian model comparison revealed that more data points are necessary to validate this correlation.

Our results regarding nitroaromatics, nitramines, and solid nitrate esters allude to the temperature of detonation being a better predictor of critical impact level than the heat of detonation. Moreover, a high ratio of bond dissociation energy to temperature of detonation indicates low impact sensitivity, whereas a small ratio suggests that the material is highly sensitive to impact. Having evaluated the predictive power of our models, we conclude that predicting impact sensitivity of energetic materials with acceptable accuracy may require inclusion of additional parameters such as hardness, crystal defects, particle size, amount of sample, heat conductivity, and heat capacity.

## Electronic supplementary material

Below is the link to the electronic supplementary material.

(PDF 485 KB)
