# Supplementary material for: Models for predicting impact sensitivity of energetic materials based on the trigger linkage hypothesis and Arrhenius kinetics
Source: J Mol Model. 2020 Mar 4;26(4):65. doi: 10.1007/s00894-019-4269-z (PMC7256078; doi:10.1007/s00894-019-4269-z)
Supplement: Supplementary file 1 — (PDF 485 KB) [file 894_2019_4269_MOESM1_ESM.pdf]

Supplementary information

Models for predicting impact sensitivity of energetic materials based on the trigger linkage hypothesis and Arrhenius kinetics

Journal of Molecular Modeling

Tomas L. Jensen, John F. Moxnes, Dennis Christensen, Erik Unneberg

Defence Systems Division, Norwegian Defence Research Establishment, P.O. Box 25, N-2027 Kjeller, Norway

Corresponding author: john-f.moxnes@ffi.no

Table 1 The nitroaromatic compounds in the Wilson et al. data set [1]. The sum formula, enthalpy of formation (ΔH_f), density, bond dissociation energy (BDE), heat of detonation (Q), temperature of detonation (T_{ex}), impact energy (I_{50}) and molecular structure

Compound	Formula	ΔH_f (s)[a] (kJ/mol)	Density[d] (g/cm^3)	BDE (kJ/mol)	Q (kJ/kg)	T_{ex} (K)	I_{50}[d] (J)	Molecular structure
Hexanitrobenzene (HNB)	C6N6O12	125.9	1.99	225.5	6816	5456	2.7	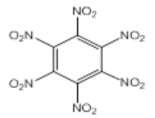
1,2,3,5,6-Pentanitrobenzene (PNB)	C6HN5O10	49.4	1.91	223.1	6578	5163	2.7	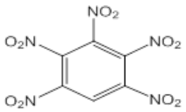
1,2,3,5-Tetranitrobenzene (TetNB)	C6H2N4O8	-43.5	1.82	226.1	5904	4528	6.9	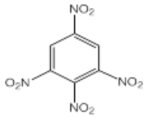
1,3,5-Trinitrobenzene (TNB)	C6H3N3O6	-47.7	1.68	284.6	5401	4026	17.4	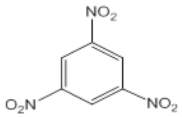

2,4,6-Trinitrophenol (Picric Acid)	C6H3N3O7	-231.2	1.77	267.6	5029	3837	15.7	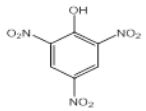
2,3,4,5,6-Pentanitroaniline (PNA)	C6H2N6O10	-388.3	1.86	213.0	5027	4123	5.4	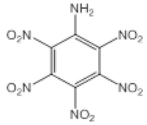
2,3,4,6-Tetranitroaniline (TetNA)	C6H3N5O8	-49.0	1.87	216.6	5656	4269	11.5	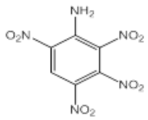
2,4,6-Trinitroaniline (TNA)	C6H4N4O6	-74.5	1.76	296.8	5077	3725	34.5	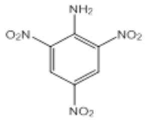
2,2',4,4',6,6'Hexanitrobiphenyl (HNBP)	C12H4N6O12	68.2	1.69	267.4	5657	4300	17.2	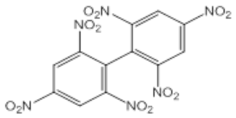
3,3'-Diamino-2,2',4,4',6,6'-hexanitrobiphenyl (DIPAM)	C12H6N8O12	-28.5	1.79	276.1	5250	3924	16.4	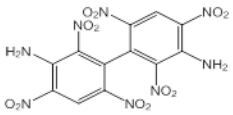
4,6-Dinitrobenzofuroxan (DNBF)	C6H2N4O6	190.0	1.76	277.5	6012	4506	18.6	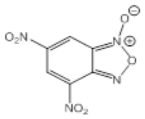
7-Amino-4,6-dinitrobenzofuroxan (ADNBF)	C6H3N5O6	153.9[b]	1.88	296.5	5640	4117	24.5	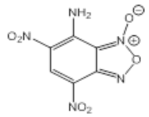
5,7-Diamino-4,6-dinitrobenzofuroxan (CL-14)	C6H4N6O6	86.0[c]	1.95	317.8	5163	3745	29.4	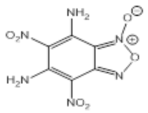

8-Amino-7-nitrobenzobisfuroxan (CL-18)	C6H2N6O6	428.9[c]	1.93	313.1	6337	4641	13.7	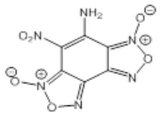
2,3,4,5,6-Pentanitrotoluene (PNT)	C7H3N5O10	-63.7[c]	1.76	226.8	6002	4608	4.4	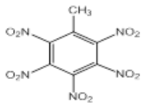
2,3,4,5-Tetranitrotoluene (2,3,4,5TetNT)	C7H4N4O8	-69.8[c]	1.71	230.1	5616	4164	3.7	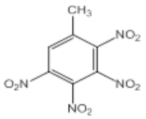
2,3,4,6-Tetranitrotoluene (2,3,4,6-TetNT)	C7H4N4O8	-69.8[c]	1.71	231.9	5616	4164	4.7	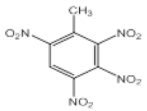
2,4,6-Trinitrotoluene (2,4,6-TNT)	C7H5N3O6	-67.1	1.67	263.5	5097	3592	24.0	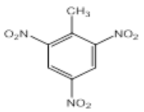
2,3,4-Trinitrotoluene (2,3,4-TNT)	C7H5N3O6	15.1	1.63	231.4	5514	3827	13.7	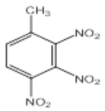
3,4,5-Trinitrotoluene (3,4,5-TNT)	C7H5N3O6	-5.0	1.63	263.9	5432	3786	26.2	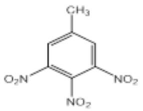
2-Amino-3,4,5,6-tetranitrotoluene (TetN-o-Tol)	C7H5N5O8	-234.7[c]	1.72	219.6	4897	3699	8.8	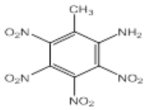
3-Amino-2,4,5,6-tetranitrotoluene (TetN-m-Tol)	C7H5N5O8	-234.7[c]	1.73	216.0	4899	3692	9.1	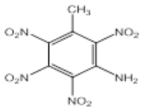

4-Amino-2,3,5,6-tetranitrotoluene (TetN-p-Tol)	C7H5N5O8	-234.7[c]	1.72	228.6	4897	3699	11.5	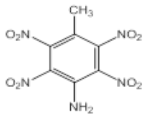
2,2',4,4',6,6'-Hexanitrodiphenylmethane (HNDPM)	C13H6N6O12	43.5[c]	1.71	254.6	5537	4055	9.6	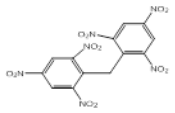

[a]Ref.[2] [b]Ref. [3] [c]Ref. [4] [d]Ref. [1]

Table 2 The nitroaromatic compounds in the Storm et al. data set [5]. The sum formula, enthalpy of formation (ΔH_f), density, bond dissociation energy (BDE), heat of detonation (Q), temperature of detonation (T_{ex}), impact energy (I_{50}) and molecular structure

Compound	Formula	ΔH_f[a] (kJ/mol)	Density[c] (g/cm^3)	BDE (kJ/mol)	Q (kJ/kg)	T_{ex} (K)	I_{50}[d] (J)	Molecular structure
Hexanitrobenzene (HNB)	C6N6O12	125.9	1.99	225.5	6816	5456	2.9	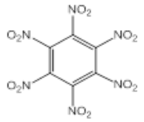
1,3,5-Trinitrobenzene (TNB)	C6H3N3O6	-47.7	1.68	284.6	5401	4026	25	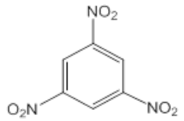
2,4,6-Trinitrophenol (Picric Acid)	C6H3N3O7	-231.2	1.77	267.6	5029	3837	21	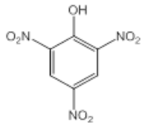
2,3,4,5,6-Pentanitroaniline (PNA)	C6H2N6O10	-388.3	1.86	213.0	5027	4123	3.7	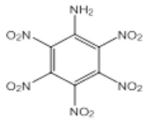

2,3,4,6-Tetranitroaniline (TetNA)	C6H3N5O8	-49.0	1.87	216.6	5656	4269	10	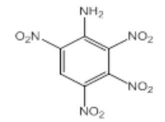
2,4,6-Trinitroaniline (TNA)	C6H4N4O6	-74.5	1.76	296.8	5077	3725	43	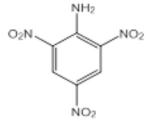
2,2',4,4',6,6'Hexanitrobiphenyl (HNBP)	C12H4N6O12	68.2	1.69	267.4	5657	4300	21	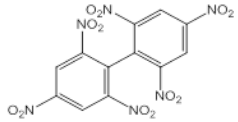
3,3'-Diamino-2,2',4,4',6,6'-hexanitrobiphenyl (DIPAM)	C12H6N8O12	-28.5	1.79	276.1	5250	3924	32	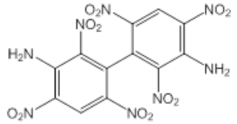
2,4,6-Trinitrotoluene (2,4,6-TNT)	C7H5N3O6	-67.1	1.67	263.5	5097	3592	39	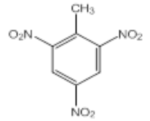
1,3-Diamino-2,4,6-trinitrobenzene (DATB)	C6H5N5O6	-98.7	1.84	310.2	4805	3486	78	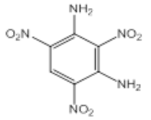
1,3,5-Triamino-2,4,6-trinitrobenzene (TATB)	C6H6N6O6	-139.7	1.94	310.2	4440	3214	>78	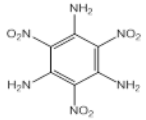
2,4,6,2',4',6'-Hexnitrodiphenylamine (HNDP)	C12H5N7O12	41.4[b]	1.64[b]	245.9	5451	4158	12	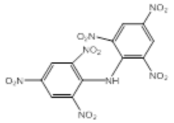
Hexanitrostilbene (HNS)	C14H6N6O12	78.2[b]	1.74[b]	243.9	5474	3981	9.6	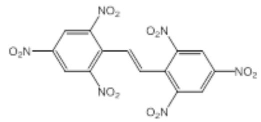

1,3-Dihydroxy-2,4,6-trinitrobenzene (Styphnic acid)	C6H3N3O8	-523.0[b]	1.83[b]	274.3	4430	3516	11	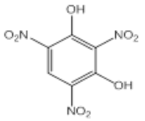
2,4,6-Trinitroanisole	C7H5N3O7	-153.2[b]	1.61[b]	244.9	5254	3780	47	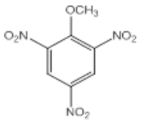
2,4,6-Trinitrobenzoic acid	C7H3N3O8	-402.9[b]	1.75[a]	269.3	4643	3639	27	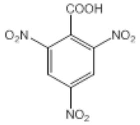
2,4,6-Trinitrocresol	C7H5N3O7	-252.3[b]	1.68[b]	255	4891	3568	47	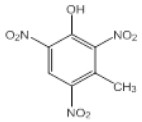

[a]Ref. [2] [b]Ref.[6] [c]Ref. [1] [d]Ref. [5]

Table 3 The nitroaromatic compounds in the Meyer et al. data set [6]. The sum formula, enthalpy of formation (ΔH_f), density, bond dissociation energy (BDE), heat of detonation (Q), temperature of detonation (T_{ex}), impact energy (I_{50}) and molecular structure

Compound	Formula	ΔH_f[a] (kJ/mol)	BDE (kJ/mol)	Density[a] (g/cm^3)	Q (kJ/kg)	T_{ex} (K)	I_{50}[a] (J)	Molecular structure
2,4,6,2',4',6'-Hexnitrodiphenylamine (HNDP)	C12H5N7O12	41.4	245.9	1.64	5451	4158	7.5	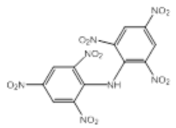
Hexanitrostilbene (HNS)	C14H6N6O12	78.2	243.9	1.74	5474	3981	5	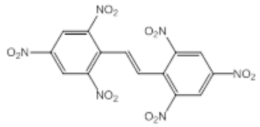

1,3-Dinitrobenzene	C6H4N2O4	-27.2	295.0	1.50	4807	3388	39	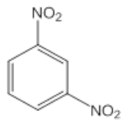
2-Amino-4,6-dinitrophenol (Picramic acid)	C6H5N3O5	-248.5	306.4	1.76[b]	4189	3026	34	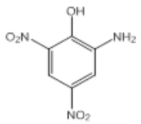
2,4,6,-Trinitrophenol (Picric acid)	C6H3N3O7	-241.6	267.6	1.77	5029	3837	7.4	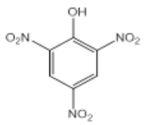
1,3-Dihydroxy-2,4,6-trinitrobenzene (Styphnic acid)	C6H3N3O8	-523.0	274.3	1.83	4430	3516	7.4	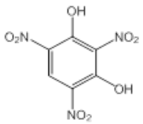
2,3,4,6-Tetranitroaniline	C6H3N5O8	-49.0	216.6	1.87	5656	4269	6	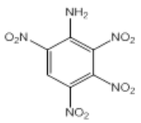
2,4,6-Trinitrotoluene (TNT)	C7H5N3O6	-67.1	263.5	1.65	5097	3592	15	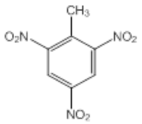
1,3,5-Triamino-2,4,6-trinitrobenzene (TATB)	C6H6N6O6	-139.7	310.2	1.93	4440	3214	50	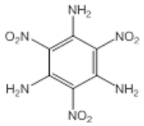
2,4,6-Trinitroaniline (TNA)	C6H4N4O6	-84.0	296.8	1.76	4978	3673	15	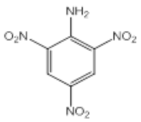
2,4,6-Trinitroanisole	C7H5N3O7	-153.2	244.9	1.61	5254	3780	20	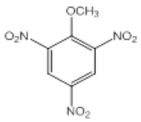

1,3,5-Trinitrobenzene	C6H3N3O6	-43.5	284.6	1.76	5630	3780	7.4	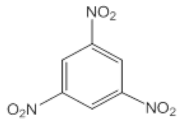
2,4,6-Trinitrobenzoic acid	C7H3N3O8	-402.9	269.3	1.75[c]	4643	3639	10	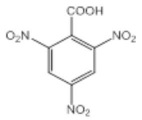
2,4,6-Trinitrocresol	C7H5N3O7	-252.3	254.9	1.68	4891	3568	12	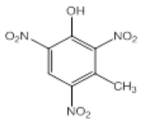
2,4,6-Trinitropyridine	C5H2N4O6	78.8	255.0	1.77	5879	4467	5.5	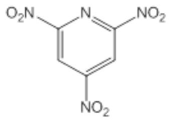
2,4,6-Trinitropyridine N-oxide	C5H2N4O7	98.7	260.4	1.86	6407	4786	2.3	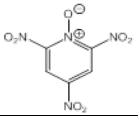

[a]Ref. [6] [b]Ref. [7] [c]Ref. [2]

Table 4 The nitramine data set [5]. The sum formula, enthalpy of formation (ΔH_f), density, bond dissociation energy (BDE), heat of detonation (Q), temperature of detonation (T_{ex}), impact energy (I_{50}) and molecular structure

Compound	Chemical Formula	ΔH_f[a] (kJ/mol)	Density (g/cm^3)	BDE (N-NO2) (kJ/mol)	BDE (C-NO2) (kJ/mol)	Q (kJ/kg)	T_{ex} (K)	I_{50}[g] (J)	Molecular structure
N,N'-Dinitro-methanediamine	C1H4N4O4	113.3	1.74[c]	220.3	-	7029	4779	3.2	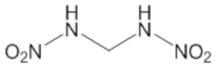

N-Nitro-N-methyl-formamide	C2H4N2O3	-80.3	1.52[d]	206.7	-	5653	3778	78.4	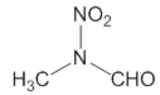
N,N'-Dinitro-1,2-ethanediamine	C2H6N4O4	-103.8[b]	1.71[b]	214.4	-	5432	3600	8.3	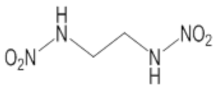
Methyl-2,2,2-trinitro-ethylnitramine	C3H5N5O8	-319.8	1.80[e]	165.1	143.3	6497	4648	2.2	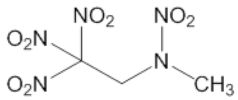
Trinitroethylnitro-guanidine	C3H5N7O8	-30.0	1.77[f]	156.5	152.6	6113	4535	3.7	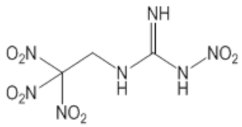
Cyclo-1,3,5-trimethylene-2,4,6-trinitramine (RDX)	C3H6N6O6	66.9[b]	1.82[b]	171.9	-	6141	4224	6.4	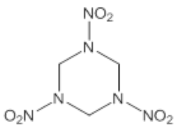
N-Methyl-N,N'-dinitro-1,2-ethanediamine	C3H8N4O4	-85.0	1.53[d]	192.5	-	5385	3435	27.9	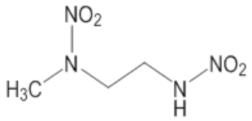
N,N'-Dinitro-N-[2-(nitramino)ethyl]-1,2-ethanediamine	C4H10N6O6	30.5	1.63[f]	182.0	-	5996	3829	9.6	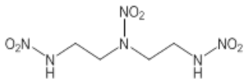

Trinitroethylcyano-methylnitramine	C4H4N6O8	-155.5	1.75[f]	154.3	138.7	5606	4308	2.7	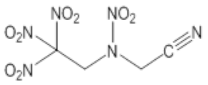
Bis-(2,2,2-trinitroethyl)-nitramine	C4H4N8O14	-353.8	1.97[f]	147.2	137.1	4373	3690	1.2	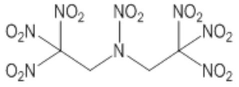
N,N'-dimethyl-N,N'-dinitrooxamide	C4H6N4O6	-305.4[b]	1.52[b]	150.4	-	4772	3534	19.4	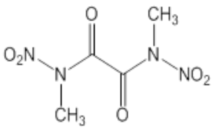
Cyclo-1,3,5,7-tetramethylene-2,4,6,8-tetranitramine (HMX)	C4H8N8O8	75.0[b]	1.96[b]	173.3	-	6036	4081	7.1	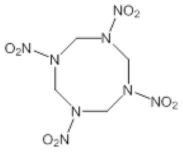
1,3,3,5,5-Pentanitropiperdine	C5H6N6O10	-210.0	1.82[f]	172.2	150.7	5949	4325	3.4	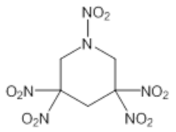
Trinitroethyl-2-methoxy-ethylnitramine	C5H9N5O9	-424.2	1.62[f]	166.7	150.7	5343	3762	10.3	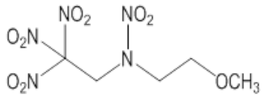
1,7-Dimethoxy-2,4,6-trinitro-2,4,6-triazaheptane	C6H14N6O8	-400.5	1.55[f]	170.5	-	5077	3308	40.7	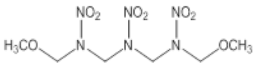

2,4,6-Trinitrophenyl-methyl-nitramine (Tetryl)	C7H5N5O8	20.0[b]	1.73[b]	130.8	258.3	5761	4183	7.8	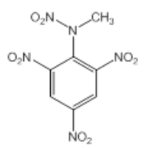
N-(2,2-Dinitrobutyl)-N-2,2-trinitro-1-butanamine	C8H14N6O10	-495.5	1.67[d]	150.6	148.5	4991	3335	19.6	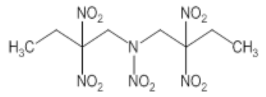
N,N'-Dinitro-N,N'-bis-(3-nitrazabutyl)-oxamide	C8H14N8O10	162.3	1.66[d]	152.8	-	6277	3980	22.1	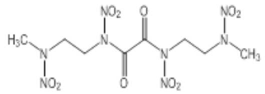
2,2,4,7,9,9-Hexanitro-4,7-diazadecane	C8H14N8O12	-438.4	1.63[d]	164.8	168	5297	3637	17.6	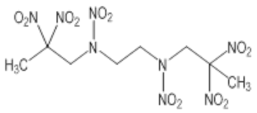

[a]Ref. [3] [b]Ref. [6] [c]Ref. [8] [d]Ref. [9] [e]Ref. [10] [f]Ref. [11] [g]Ref. [5]

Table 5 The nitrate ester data set [6]. The sum formula, enthalpy of formation (ΔH_f), density, bond dissociation energy (BDE), heat of detonation (Q), temperature of detonation (T_{ex}), impact energy (I_{50}) and molecular structure

Compound	Formula	ΔH_f[a] (kJ/mol)	Density[a] (g/cm^3)	BDE (kJ/mol)	Q (kJ/kg)	T_{ex} (K)	I_{50}[a] (J)	Molecular structure
Dinitrophenoxy-ethylnitrate	C8H7N3O8	-292.8	1.60	161.6	5058	3573	20	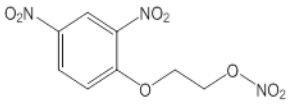
Dioxyethylnitramine dinitrate (DINA)	C4H8N4O8	-275.7	1.49	160.1	5968	4139	6	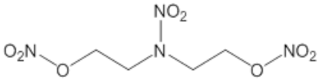

Dipentaerythritol hexanitrate (DIPEHN)	C10H16N6O19	-978.6	1.63	159.6	5754	3985	4	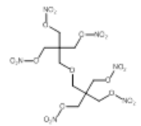
Erythritol tetranitrate	C4H6N4O12	-502.5[b]	1.60	144.1	5709	4393	2	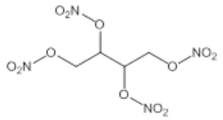
Mannitol hexanitrate (MHN)	C6H8N6O18	-675.6	1.60	147.9	5801	4470	0.8	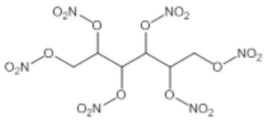
Pentaerythriol tetranitrate (PETN)	C5H8N4O12	-538.8	1.76	151.9	6203	4337	3	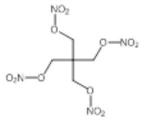
Trinitrophenoxyethylnitrate	C8H6N4O10	-260.3	1.68	158.7	5495	3996	7.9	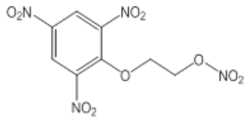
Nitroglycerine (NG)	C3H5N3O9	-370.6	1.59	153.0	6087	4541	0.2	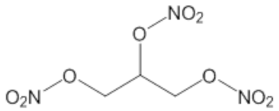
Methyltrimethylol-methanetrinitrate (TMETN)	C5H9N3O9	-425.0	1.46	154.4	5840	3891	0.2	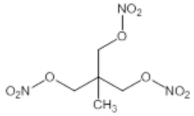
Butanetriol-trinitrate (BTTN)	C4H7N3O9	-405.9	1.52	149.1	6119	4330	1	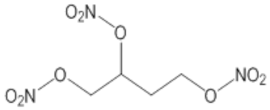
Diethyleneglycol dinitrate (DEGDN)	C4H8N2O7	-436.7	1.38	164.3	5495	3728	0.1	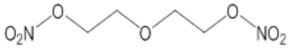

Methylnitrate	C1H3N1O3	-155.9	1.22	175.3	6310	4418	0.2	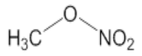
Nitroglycol (EGDN)	C2H4N2O6	-242.7	1.48	160.9	6424	4668	0.2	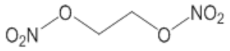
Triethyleneglycol dinitrate (TEGDN)	C6H12N2O8	-628.8	1.34	165.3	4918	3218	12.7	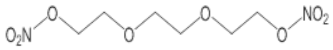
Trimethylolnitromethane trinitrate (NIBTN)	C4H6N4O11	-228.1	1.68	149.9	6815	4946	2	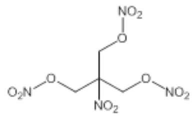

[a]Ref. [6] [b]Ref. [2]

References

1. Wilson WS, Bliss DE, Christian SL (1990) Explosive Properties of Polynitroaromatics. Naval Weapons Center: China Lake, California
2. (1997) Data Base of Thermochemical Data. Fraunhofer Institut für Chemische Technologie
3. Keshavarz MH (2011) Prediction of the condensed phase heat of formation of energetic compounds. Journal of Hazardous Materials **190**(1):330-344
4. Keshavarz MH (2009) Predicting condensed phase heat of formation of nitroaromatic compounds. Journal of Hazardous Materials **169**(1):890-900
5. Storm CB, Stine JR, Kramer JF (1990) Sensitivity Relationships in Energetic Materials. In: Chemistry and Physics of Energetic Materials, Springer, pp 605-639
6. Meyer R, Köhler J, Homburg A (2007) Explosives, 6th edn. Wiley-VCH Verlag GmbH, Weinheim
7. (2007) ICT Database for Thermochemical values (Demo Version). Fraunhofer Institut fur Chemische Technologie, Pfinztal
8. Pristera F et al (1960) Analysis of explosives by using infrared spectroscopy. Anal. Chem. **32**:495-508.
9. Rice BM, Hare JJ, Byrd EF (2007) Accurate Predictions of Crystal Densities Using Quantum Mechanical Molecular Volumes. The Journal of Physical Chemistry A **111**(42):10,874-10,879

10. Chen SP et al (2003) Kinetics of the exothermic decomposition reaction of N-methyl-N-nitro-2,2,2-trinitroethanamine. Chin. J. Chem. **21**(11):1,419-1,421
11. Adolph HG, Holden JR, Chicra DA (1981) Relationships Between the Impact Sensitivity of High Energy Compounds and Some Molecular Properties Which Determine Their Performance. Naval Surface Weapons Center: Silver Spring, Maryland
